# Trauma‐Informed Principles in Practice: A Mixed‐Method Study of Co‐Producing Systems Change With People Who Have Experienced Multiple Disadvantage

**DOI:** 10.1111/hex.70472

**Published:** 2025-11-02

**Authors:** Tracey Stone, Emily Eyles, Thomas Traub, Jason Burrowes, Rebecca Halsley, S.J.M., Joanna Gillam, Maria Theresa Redaniel, Sabi Redwood, Corrado Totti, Tania Smith, Michelle Farr

**Affiliations:** ^1^ The National Institute for Health and Care Research Applied Research Collaboration West (NIHR ARC West) at University Hospitals Bristol and Weston NHS Foundation Trust Bristol UK; ^2^ Population Health Sciences, Bristol Medical School University of Bristol Bristol UK; ^3^ Independent Futures Changing Futures Bristol UK; ^4^ National Cancer Registry Ireland Cork Ireland

**Keywords:** co‐production, multiple disadvantage, systems change, trauma‐informed practice

## Abstract

**Background:**

Health and social care services increasingly recognise the value of involving people with lived experience in service design and delivery. For people who have experienced multiple disadvantage (combinations of homelessness, mental ill health, addiction, involvement with the criminal justice systems or domestic violence/abuse), participating in professional settings may be challenging and can risk re‐traumatisation and greater disillusionment. However, gaining confidence and competency to do this offers opportunities for personal and professional development, contributing to meaningful change. It is necessary that individuals with lived experience can engage safely and effectively in these settings. This study analyses how an organisation of people who have experienced multiple disadvantage, Independent Futures (IF), enabled co‐production within services and systems, to understand how people can be best supported and how involvement impacts them.

**Methods:**

Sixteen IF members and three IF staff participated in semi‐structured interviews. A staff survey, at two time points, investigated how employees from Changing Futures partner organisations perceived their ability to embed co‐production within services, with 147 responses. Internal documentation was analysed to illustrate the diversity of co‐production work that IF contributed to.

**Results:**

Putting trauma‐informed principles into practice facilitated personal growth, improved confidence and some work skills for IF members, who contributed to 65 different workstreams. However, embedding co‐production into wider services and systems proved challenging. Staff survey comments highlighted obstacles related to resources, time and hierarchical cultures.

**Conclusion:**

Lived experience organisations can model trauma‐informed practice and influence systems. Embedding trauma‐informed principles requires flexibility, openness and willingness that is sustainable only when everyone adopts and commits to these principles. Any evidence of tokenism destroys trust and undermines the endeavour.

**Public Contribution Statement:**

This study was co‐produced with people with lived experience of multiple disadvantage and staff participants. Two lived experience IF members were involved in: developing the funding bid, designing the research including designing the staff survey, developing interview topic guides, commenting on information sheets and developing interview arrangements to ensure comfort and safety of IF members. Four lived experience IF members and three members of staff contributed to writing the paper, including reviewing key literature, refining the analysis and developing the discussion and conclusion.

## 1 Background

### 1.1 Multiple disadvantage (MD) and Trauma‐Informed (TI) Approaches

There is increasing policy attention on people who experience MD, defined as individuals who have faced combinations of homelessness, drug and alcohol problems, mental ill health, domestic violence and abuse, or contact with the criminal justice system [1, 2]. People who have experienced MD often have backgrounds of adverse childhood experiences, adversity or trauma [3, 4]. Trauma results from events that are physically or emotionally harmful or life threatening [5, 6]. Facing homelessness or violence or abuse are inherently traumatic, which can compound earlier adverse childhood experiences and traumas [4]. Trauma‐informed approaches have been identified as critical in addressing the needs of people with MD, and there is some evidence of positive outcomes [7]. Trauma‐informed approaches are strengths‐based practices which recognise the impact of trauma, ensuring physical, psychological and emotional safety and opportunities to empower people. They incorporate TI principles (TIPs) of safety, trustworthiness and transparency, peer support, collaboration, empowerment/choice, and cultural, historical and gender issues/inclusivity [5]. The implementation domains that support the inclusion of these TIPs into organisational practice include governance and leadership, policy, physical environment, involvement of people with lived experience, cross‐sector collaboration, assessment and treatment services, training and workforce development, monitoring and quality assurance, financing and evaluation [5]. TIPs need to be embedded within these implementation domains to support the development of a TI culture.

Current debates about MD are motivated by concerns that policies are not working [1]. For example, homelessness figures are rising [8] and more prison leavers are ending up homeless [9]. Women who've experienced sexual violence have reported that the social security system can worsen survivors' mental and physical health conditions and replay abusive interactions [10]. These examples can be seen as public value failures [11], or value destruction [12, 13], where public services may have a negative impact on service users. It has been highlighted that policymakers are often distant from the realities of people's lives [14] and so may not have the necessary knowledge and insights to create effective policies. Thus, people with lived experience of MD need to be involved in policy and service design [14, 15]. Work is only recently emerging on co‐production's applicability to people with lived experience of MD [16, 17, 18] and how trauma‐informed adaptations might be required to respond to the needs of people experiencing MD [19, 20]. This article contributes to this emerging evidence base, analysing how TIPs operate in practice within a lived experience organisation, Independent Futures (IF), that aimed to change MD services and systems, analysing the process and outcomes of IF's work, alongside staff perspectives on co‐production. It adds to theoretical developments, illustrating how TI implementation domains can support co‐production to be more holistically embedded, so that co‐production is embedded from policy to practice, training and development to monitoring and evaluation.

### 1.2 Co‐Production and MD

Whilst there is considerable conceptual diversity of co‐production [21, 22], a common theme is how co‐production acknowledges the inherent skills and resources of service users, to ensure everyone works together with the intention of producing more equitable partnerships within services [23]. Co‐production can occur through all stages of a service process (planning, commissioning, service provision and monitoring) [24]. This paper focuses on co‐production, where people with lived experience of MD are involved in work with staff to improve health, social care and other public services through strategy, policy, planning, service design and provision, and evaluation and research. We use the term people with lived experience to describe those who have experienced or may be experiencing MD. Whilst historic experiences of trauma/MD can stay with a person many years after they have occurred, there are debates around the extent to which historic service experiences are relevant to developing current services [25], as services may have changed since that person was using them. However, working alongside people with lived experience of MD can enable services to offer more appropriate, trauma‐informed support [26].

Co‐production literature often assumes that active involvement of service users can improve public value [11]. However, there are barriers to co‐production for people with lived experience of MD that can lead to marginalisation even within collaborative processes [16, 23]. Table 1 outlines some of these barriers and identified facilitators, and how applying TIPs through the implementation domains may support this study, building on literature about co‐production with disadvantaged groups [19, 32, 34, 35].

**Table 1:** Evidence on barriers and facilitators to co‐production for people who experience multiple disadvantage and their relevance to trauma‐informed principles.

Barriers (and references)	Facilitators	Related TIPs and implementation domains [5]
Skills and abilities [27]. Staff might not have the time, expertise, facilitation skills or inclination to involve people with lived experience of MD, or may lack confidence in their own or people with lived experience's abilities [17]. Training for people with lived experience can be limited, with few progression opportunities for career development [2].	Dedicated resources and flexibility are needed to develop inclusive co‐production processes, so that a diverse range of people can get meaningfully involved on their own terms [2, 28]. Skills development and training for people with lived experience are important to engage effectively in co‐production opportunities [27]. However, there is limited evidence whether involvement in co‐production could be a pathway to such education, training and employment.	Collaboration Empowerment Cultural, historical and gender issues *Training and workforce development* *Financing*
Services still routinely operate top‐down hierarchical approaches that can lead to tokenism and disempowerment [17], intimidating formats for involvement [27] or create a clash of cultures because service user involvement is more democratic [29]. Equitable power sharing within these contexts can be challenging [30].	Organisational enablers include: a shared vision, distributed leadership, management involvement in co‐production, staff autonomy to make decisions and flat organisational hierarchies that value empowerment [18]. Relational and asset‐based approaches can support empowerment of people with lived experience [31].	Collaboration Empowerment *Governance and leadership* *Policy* *Physical environment*
People with experience of MD need motivation [20, 27] and resilience to develop new skills and ways of working, whilst managing their own mental health, addiction or housing issues [17].	Issues can vary between individuals, and some may need more support than others [29].	Empowerment *Assessment and treatment services*
Developing trust with people who have been let down by services and historically difficult relationships [17, 18, 20]. Mismatched expectations and different perspectives may disillusion people with lived experience [27].	Trust can be facilitated through joint interests, shared concerns and the redistribution of power and responsibility.	Trustworthiness and transparency *Governance and leadership* *Training and workforce development*
Balances between safety and empowerment can be difficult to manage [19].	Ensuring the appropriate level of autonomy is important and may be different for different people [27, 32]	Safety and empowerment
Emotional challenges [27] and repeated sharing of experiences can activate trauma and flashbacks, while risking relapse [25]. Where there are difficulties in one individual′s recovery, it may lead to challenges or impact others [29].	Peer support created through lived experience groups can be important to members [19, 29]. Opportunities can prove beneficial and promote long‐term recovery [15] [33].	Peer support

### 1.3 Changing Futures, Co‐Production and the Lived Experience Organisation, Independent Futures (IF)

**Figure 1:**
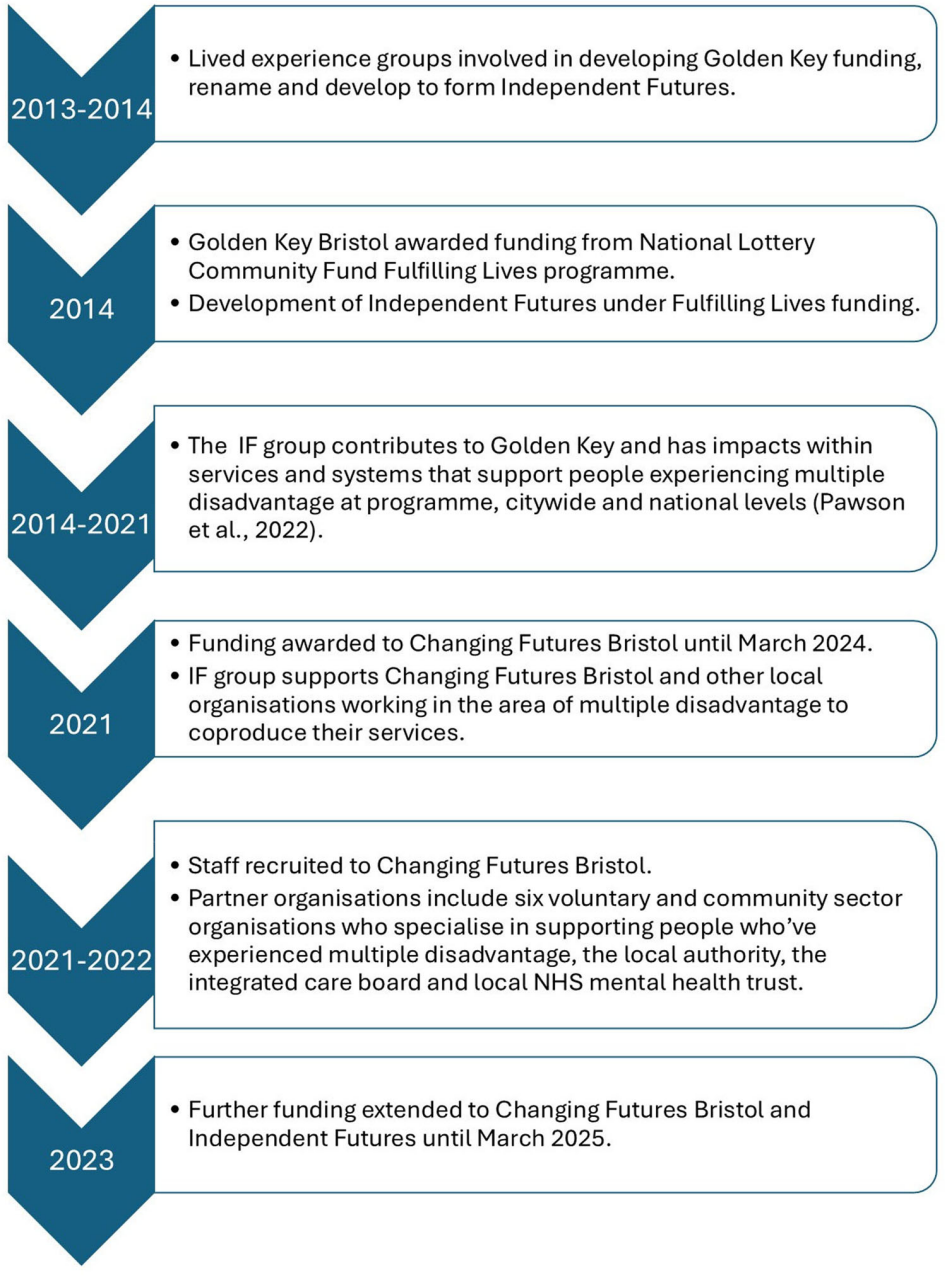
Timeline of Golden Key, Changing Futures and Independent Futures in Bristol.

The Changing Futures programme aimed to support innovative approaches for people who've experienced MD [2] through a £77 million government and National Lottery Community Fund initiative running in 15 areas across England. IF was one of several organisations involved in Changing Futures Bristol (CFB), as a lived experience group that has contributed to service development since 2013. Initially IF was funded through Fulfilling Lives National Lottery funding, known locally as Golden Key [36, 37]. IF's purpose was to support the development of services for people with MD, previously led by Golden Key and then CFB, and also those provided by local voluntary and statutory organisations [29]. Previously under the Golden Key programme, contributions from IF had led to impacts at a programme, city‐wide and national levels [29]. This article picks up on the activities of IF under Changing Futures funding from 2021 to 2024 (see Figure 1).

## 2 Methods

### 2.1 Research Aims

This independently funded evaluation of CFB ran from January 2022 to June 2024 with collaborative write‐up continuing into 2025. This article focuses on research objectives to:

1. Analyse how organisations can support people who've experienced MD through different trauma‐informed and co‐production approaches.
2. Understand how to best support lived experience representatives and how involvement impacts them.
3. Assess what skills and resources are needed to embed trauma‐informed and co‐production approaches.
4. Analyse the impacts of implementing trauma‐informed and co‐production approaches on lived experience representatives.

A further article explores how trauma‐informed approaches were implemented from the perspectives of staff through interviews and a survey [38]. The University of Bristol Faculty of Health Sciences Research Ethics Committee approved the research, reference 12277. The local authority involved also approved the study, BNSSG ICB reference: 2022‐087 and BCC reference: 2022‐020.

### 2.2 Participatory Research Approach

This evaluation followed ‘co‐producing research′ guidance wherever possible [39], guided by participatory action research and co‐operative inquiry where research is done with people rather than on them [40, 41]. Two lived experience IF members were involved in developing the funding bid, designing the research including the staff survey, developing interview topic guides, commenting on information sheets and developing interview arrangements to ensure comfort and safety of IF members, and two more joined to develop analysis and write‐up.

Initial research development meetings with two IF members were held in informal community venues over food. Coming out of the Covid‐19 pandemic, the return to face‐to‐face meetings and opportunity to build relationships and trust through informal meetings was important. As the research was designed, the research team, IF staff and IF members discussed whether it was inappropriate for IF members to be involved in co‐interviewing and co‐analysing interview data, as this would mean interviewing friends, colleagues and peers. To maintain confidentiality, we agreed that interviews would be conducted by a university researcher only and only the university research team would have access to the transcripts and conduct the initial analysis. The university researcher had over 20 years' experience of interviewing service users in social and health inequalities, and interviews were conducted with care, consideration and in line with TIPs to avoid re‐traumatisation, for example, not asking questions about lived experiences but focusing on IF activities.

### 2.3 Qualitative Data Collection and Analysis

The study was introduced to the wider IF membership at a weekly meeting, with a verbal invitation to be interviewed. This included a short video for IF members not attending the meeting, where the researchers explained the study and what participation would entail. Participant information documents were handed to people at the meeting (and emailed), and where requested, the topic guide was shared with potential participants. Participants could choose either in‐person interviews in a private meeting room that participants were familiar with, alternatively online or telephone interviews, at a date and time to suit them. Participants gave written/verbal (recorded for online/phone) consent before the interview began. Post‐interview support was arranged if needed, with an IF staff member being on hand if a member wanted to de‐brief after the conversation. IF members were offered £20 to compensate them for their time, following IF reimbursement mechanisms. Further invitations to take part in an interview were repeated several months later at a weekly meeting, where initial findings from the first eight interviews were shared for discussion with the group. Due to the intervening recruitment drive, 12 new members had joined IF and 8 of these agreed to be interviewed. Sixteen interviews took place with IF members, and three with IF staff, with a total of nineteen interviews. Interviews lasted between 22 and 50 min (average 37 min) and were audio recorded, transcribed, checked for accuracy, anonymised and imported into NVivo for coding. Interviews were double coded, and a coding frame was agreed by the first and last authors, initially through inductive and thematic analysis, which was informed by TIPs [5]. Further deductive analysis explored how IF's activities supported different implementation domains of TI practice [5]. The first author coded all further transcripts, with coding discussed between first and last authors as analysis progressed. The topic guide was developed iteratively over time to reflect insights arising from preliminary analysis. Themes arising from all interview data were discussed and developed with the full IF member group. Anonymised versions were shared to summarise findings. Internal documentation was analysed by an IF staff member to catalogue all activities and outcomes that IF had worked on. This documentary analysis ensured accuracy and breadth when reporting activities, rather than solely relying on interview accounts, where participants may not mention all the different activities they had been involved in.

### 2.4 Quantitative Data Collection and Analysis

A staff survey was co‐designed with CFB staff and IF members, using existing survey measures where possible, alongside programme‐specific questions. Existing standardised surveys included questions on trauma‐informed approaches [42], co‐production [43], staff well‐being [44] and working conditions [45], alongside free text boxes on these different dimensions. An invitation to take part in the survey, hosted by the university's secure survey system REDCap [46], was sent to relevant staff across CFB voluntary sector partners and local authority adult social care teams (as detailed in Figure 1), by CF partner leads who held staff email lists (December–March 2023—time point 1), and was repeated a year later (December 2023 to March 2024—time point 2). As there was some staff turnover, staff who had not previously participated were invited to complete a survey at time point 2. New participants in the second survey were included in the second time point for the analysis. Data were analysed using Stata 17.0 and R4.3.1 and descriptive statistics were calculated. This article reports on the co‐production audit questions and free text responses. Other survey results are reported separately [38], alongside a broader range of staff interviews (*n* = 23).

### 2.5 Participatory Analysis and Write‐Up

Whilst two IF members were involved in designing the research, two further members joined the analysis and write‐up group. One joined the team to present initial findings at two national academic conferences. Another joined the write‐up team which met regularly over 7 months. Members' involvement was supported by IF staff. The write‐up team reviewed literature, developed each article section together once the analysis was anonymised, and reviewed and edited the full manuscript. Lived experience co‐authors were very keen to make language within the article as accessible as possible, with a view to encourage a wider readership beyond academia.

## 3 Findings

The following sections demonstrate key findings from interviews, followed by the staff survey. IF membership is described, with an overview of the recruitment process and IF members' reflections on why they joined. Illustration of how IF's working practices were influenced by TIPs is shown through an analysis of group functioning and members' training and personal development. The work that IF members undertook and the impact of this is summarised, before presenting staff perspectives of co‐production from the survey.

### 3.1 IF Membership, Recruitment and Reflections on Joining IF

**Figure 2:**
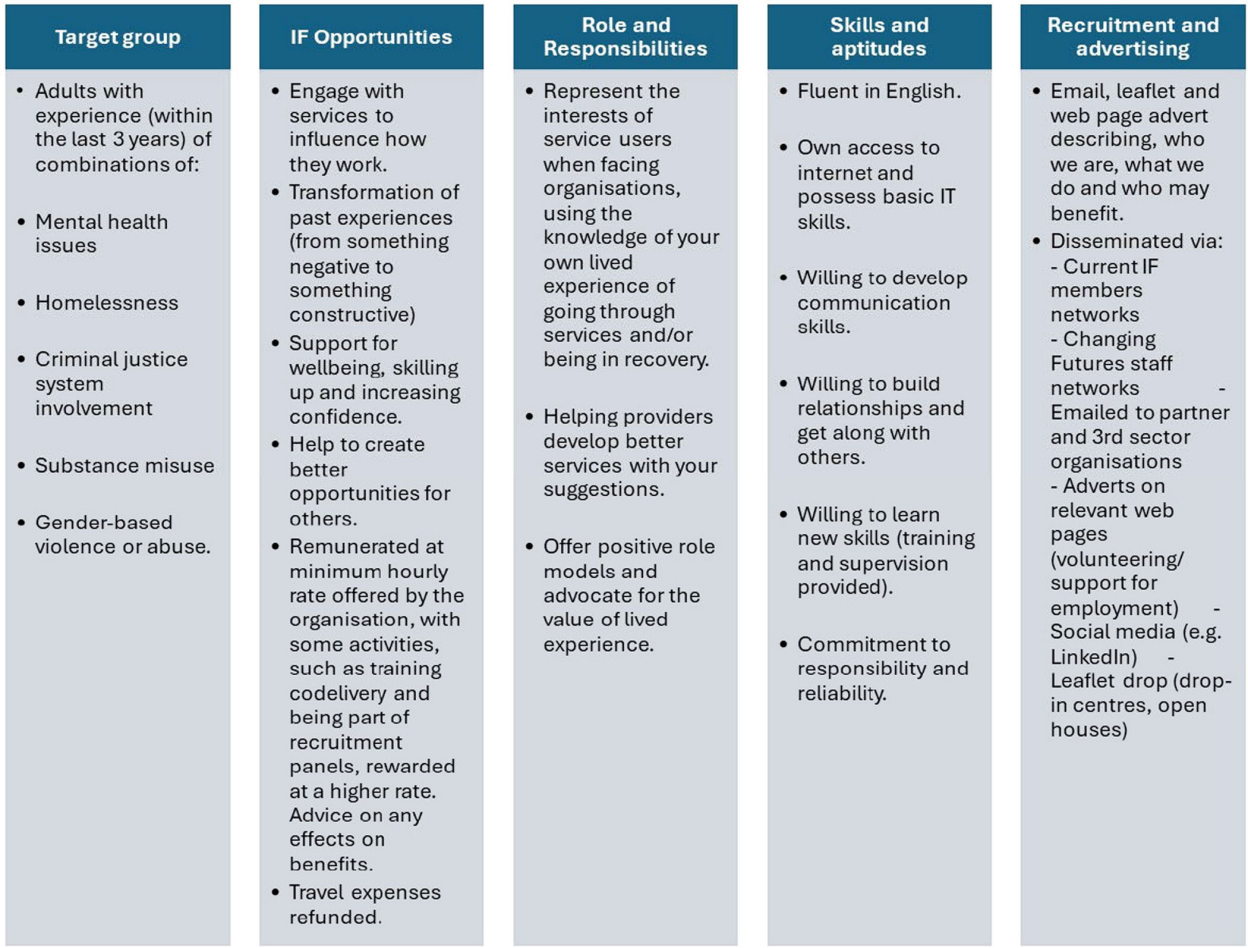
Independent Futures role description and recruitment.

In the shift to Changing Futures funding (Figure 1) it was seen that a greater diversity of IF members was needed to represent clients. A recruitment drive (Figure 2) brought the total number of IF members to 17. Following this recruitment, IF had similar numbers of male and female identifying members, as well as some non‐binary members. Ethnicities included White British and European, Black African/Caribbean and Indian heritage. IF members had lived experience of the diversity of MD and ages ranged from 20s to 60s.

Introductory recruitment meetings took place one‐to‐one in a neutral location, usually a local café to avoid anxiety that may have occurred in an office setting and to minimise power imbalances. Places and location to sit were chosen by potential members themselves to make the situation as relaxed as possible. The purpose was to informally meet in person, not a formal interview where potentially confidential information could be shared. The conversation was mainly about CFB and IF and how lived experience voices were essential in shaping systems change. TIPs of ‘safety’ and ‘choice’ were demonstrated by being notified that there were many ways of engaging to suit them, they did not have to speak and could contribute in different ways, and that self‐care takes priority over all else. During the recruitment process, no one was rejected, avoiding a potentially re‐traumatising event. Rather, the offer remained open for ‘when they were ready’, demonstrating attention to inclusivity as a TIP.

All members interviewed said that the opportunity to improve services was a reason for engaging with IF. This was particularly highlighted by longer‐term members who were usually more stable in their recovery and personal/professional development, with greater experience of engaging with service partners. Nine of the 16 (mainly newer members) said that this was a secondary consideration that developed increasingly over time, after the initial need to seek contact, interaction and engagement with others to support their recovery. For some members, transforming personal trauma into influencing services to benefit others generated a new‐found sense of agency (Table 2, Quote Q1).

**Table 2:** Quotes about joining IF, flexible engagement and demonstrations of trauma‐informed principles.

No.	Quote text	Trauma‐informed principles
Q1	‘*They were asking me and all my failures in life were like, standing ovation! That I am very good for the job, you know what I mean?…. It gave me really a lot of confidence because all my failures, all my past experiences, now I can use to help other people’.* (IF3)	Empowerment/choice
Q2	‘*(T)here was a big emphasis on “you don′t need to do anything,” “everything is optional, you can stop at any point.” There′s no commitments…. So at the time, continually less now, I was [sighs] if I had gone to a single meeting lasting an hour, it would′ve taken me two days to recover from it … you recover for the next time but then you adapt to it and then you slowly, slowly build’.* (IF13)	Safety Empowerment/choice
*Q3*	‘*(I)f there′s one thing that I think is important when you involve people with lived experience is to have that structure because we′re used to chaos and doing it on the fly and just reacting. The structure allows people to take stock and take things serious(ly)’.* (IF5)	Safety (and boundaries)
Q4	‘*(T)he idea of mutual relationships, safety, not just safety for people with lived experience, everyone′s safety … groups need to be made accessible to people with lived experience, but lived experience (members) also are not expected to threaten professionals or to blame them or to diminish what they are doing’.* (Staff14) ‘*I also show a little bit of vulnerability, and show my, my underbelly, so that you know, don′t think of me as this example of perfection, far from it, also to indicate that progress is achievable, it doesn′t matter what your background is, if you′re dedicated enough, if you′re committed, if you′re focused, you can get on with your life and get to places where you want to be’.* (Staff14)	Safety Collaboration Trustworthiness and transparency Peer support
Q5	‘*I think in the past … I always thought that professionals knew better than me … all the consultants were right. Any worker who I was working with I kind of thought they knew everything and what I′ve learnt is, no they don′t. Like professionals are just as fallible as anyone else and in reality, I know myself better than they know me because I am living my life and that′s not to say that professionals can′t be helpful and that they don′t have various insights and different knowledge that is helpful but it′s very damaging as a client to always believe that others are right and you′re wrong. So I think that has changed over time as well and actually that has really helped me in services because I can challenge stuff and kind of go, that′s not okay. Rather than kind of being a client doormat’*	Empowerment
Q6	‘*You have got plenty of opportunities in which you can actually feedback and be able to see weeks forward how that feedback that you′ve contributed has been able to change the things that needed to be changed’.* (IF9)	Trustworthiness and transparency

### 3.2 Flexibility, Accountability and Prioritising Self‐Care

At meetings, IF members could attend in person or online; have camera/microphone off or on; speak or not speak as they feel comfortable; and could email comments after the meeting. Prioritising personal well‐being helped to manage the effects of previous trauma and was essential to continued engagement in many cases (Table 2, Q2).

Alongside flexibility and accountability, defining and reinforcing boundaries was important. Two interviewees described how this considerate but firm approach was significant, where IF members' lives might otherwise be unstructured (Table 2, Q3). Two staff were available before and after meetings and at other times as required, for 17 members. This provided support to members who may be triggered by discussion topics, may want to offer thoughts or observations on the agenda without speaking in the meeting, or face other challenges in their lives that they wanted to discuss.

### 3.3 Demonstrations of Trauma‐Informed Practice

TIPs were demonstrated reliably through actions, interactions and decision‐making. Posters in the office, and on meeting room walls, reminded staff and IF members of the importance of safety, trust, choice, collaboration, empowerment and cultural considerations. This applied to all engagement and support of IF members but also, importantly, to how IF staff worked together. Traditional power dynamics between staff and IF members were addressed in early training sessions, to engender trust and mutual respect (Table 2, Q4).

Respect and self‐care were demonstrated by staff in setting and enforcing personal boundaries, which served as examples of personal conduct to members. This contrasted with previous negative experiences of services where people may have felt blamed, shamed or belittled (Table 2, Q5).

IF members reported examples of staff being open to feedback and action being taken on that information. Where a request could not be met, or met in the way suggested, this was explained and discussed either in the group meeting or individually as appropriate (Table 2, Q6).

### 3.4 Training, Group Functioning and Personal Development

Training served several purposes. It provided opportunities to navigate power dynamics between trainer (authority) and trainee (IF member), who may have had negative expectations of this dynamic because of their lived experience. Some new IF members were unfamiliar with ‘training’ in any form. This was acknowledged and supported. The impact of this induction and training approach was noticed within the first weeks of membership (Table 3, Q7).

**Table 3:** Training, group functioning and personal development quotes.

No.	Quote text	Trauma‐informed principles
Q7	‘*I would normally be mute and not say a word heart racing, feeling completely and utterly awkward, but I think if I didn′t understand something I asked a question which I would never normally have done. I sort of put that down to the way I was brought in’.* (IF11)	Safety
Q8	‘*In one of the inductions someone′s oversharing so I had half the room shaking, so I knew from the next induction I need to put in place, what′s the risk of oversharing, what′s the cost of it’.* (Staff13)	Safety Empowerment and choice
Q9	‘*It′s just working in that zone which is just right outside of our comfort so that we can grow professionally and personally to make it our comfort zone….’* (Staff13)	Empowerment and choice Safety
Q10	‘*(F)or me, there′s a dynamic there that is special because we all have similarities of issues. What you get from that group is undefinable. It′s a support that is different to any other type of support you would get’.* (IF5)	Peer support
Q11	‘*I haven′t got time for those toxic people anymore so it′s like I′ve regained that independence, I′ve broke free from that now’.* (IF15)	Empowerment and choice
Q12	‘*Building a cohesive team … it′s beneficial to everyone … I think they′re quite good at doing that but then that begs the question of are they leaving some people out that could be … say they were volatile but maybe they like being included, that′s a question you could ask’.* (IF13)	Inclusivity Safety

IF members were supported to identify their own training needs, which appeared to equip IF members to contribute effectively to IF meetings and workstreams. Training covered practical skills such as IT, self‐awareness approaches, advocacy for self and others, boundary management, for example whether, when and how much to share one's lived experience, avoiding oversharing and the possible reactivation of trauma. Given the collective trauma history within IF, the likelihood of activating distress was high (Table 3, Q8).

Interviewees involved in IF for longer reflected on how IF had evolved from being the ‘Wild West’ (IF5) to a more stable, structured environment with clear boundaries. The ability to have constructive engagement with authority figures was an important transferable skill. Coping mechanisms learned from IF training on personal boundaries, self‐awareness, listening and self‐protection were evident in interview responses. However, members also spoke of examples of difficult behaviour in meetings that were sometimes hard to manage or respond to. IF meetings that were particularly uncomfortable were followed by a debriefing with group members before they left, with one‐to‐one support available.

Skills acquisition extended into weekly IF member meetings where some members took on a facilitation role. IF members were supported to lead co‐production workshop training. Again, flexibility in how members contributed broadened inclusion and development opportunities; some members delivered training whilst another member contributed significantly to developing materials for events (Table 3, Q9).

A strong sense of group loyalty and mutual support existed between group members (Table 3, Q10). Some described improvements in self‐worth and ambition, based on their experiences and accomplishments with IF (Table 3, Q11). Recovery was supported within IF as described above, but a degree of consistent stability was required for work with other agencies. The group included members at different stages of recovery; however, the inclusion of a broader spectrum of people who may be earlier in their recovery could have had an impact on dynamics (Table 3, Q12). This also relates to questions of representativeness, where lived experience members tended to be slightly further away from being current service users, according to their levels of recovery. IF members were rarely involved in service improvements of services that they currently used.

### 3.5 Workstreams and Impact

**Figure 3:**
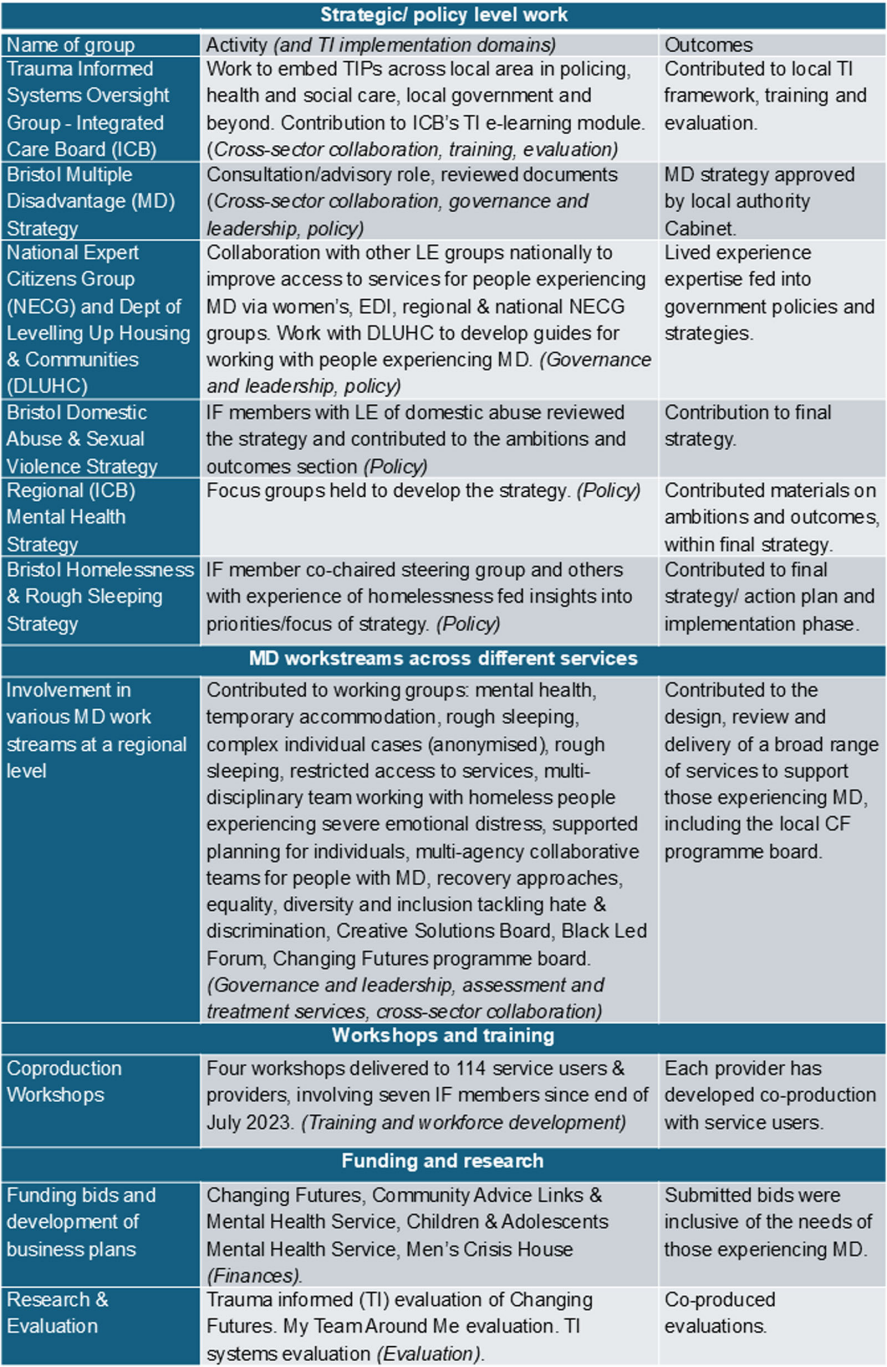
Activities of Independent Futures through Changing Futures funding with associated TI implementation domains (middle column).

IF members were involved 65 workstreams within and beyond the Changing Futures programme, across all MD sectors. Twenty‐nine of these workstreams involved regular meetings, whilst the remaining were more ad hoc, one‐off involvement opportunities. These included strategies/policies; consultation/advisory work; co‐planning of proposals; co‐design/co‐delivery of services/interventions; operational work (e.g., interviewing staff/client selection panels); reviewing documents for accessibility and language; client facing work; and research and evaluation. Figure 3 focuses on some of those regular workstreams, illustrating a range of activities and contributions to outcomes, with the middle column illustrating how they relate to the TI implementation domains.

Some IF members received individual informal feedback on their contributions to work with other agencies at the time. Where this happened, it was positive and appreciated. The possibility of tokenism was raised by two participants, and formal feedback was seen as a potential mechanism to provide reassurance that this was not the case (Table 4, Q13).

**Table 4:** Workstreams and impact quotes.

No.	Quote text	Trauma‐informed principles *(Implementation domains where appropriate)*
Q13	‘*I don′t want to be part of something just so they can say that look we have got this group as part of this. That just makes you feel as though you are just a box that has been ticked to satisfy something, you know?’* (IF14)	Trustworthiness and transparency
Q14	‘*I sit among really senior management of organisations, not all engaged, but the ones that do, I have terrible experiences from their agencies … when you′re in the system and when you′re a service user you′re like here like low and they′re here but this opportunity…. I′m like me and you are the same … we′re the same, I′m sitting here just like you are and I′ve an opportunity here if I want to be critical of you and you have to take it on board because all of the other senior members of staff are looking at you’* (IF2)	Empowerment Collaboration *(Lived experience involvement in governance and leadership, policy)*
Q15	‘*I always thought that professionals knew better than me…. What I′ve learnt is, no they don′t. Like professionals are just as fallible as anyone else and in reality, I know myself better than they know me because I am living my life’* (IF7)	Empowerment Collaboration
Q16	‘*The simple solution to this would be a time limited membership, so to recruit a new IF member on a 12‐18 months basis … plan for the whole duration of the membership and agree to development plan with members … to look at how IF can help them in their career, in their recovery, in a way that is goal oriented and time bound’* (Staff 14)	Empowerment Trustworthiness and transparency

IF members were rightly proud of the contribution they made locally and sometimes, nationally. The opportunity to sit and discuss key policy issues was potentially empowering and enabling and many members spoke of the personal impact that IF involvement had had on them (Table 4, Q14/Q15).

Some staff viewed outcomes as successful when IF members had acquired sufficient skills and confidence to be independent and move on to employment or education. In contrast a few IF members expressed a degree of frustration that they were unable to do more IF work with greater breadth and influence and wanted to stay within IF. However, staff were concerned that IF members should not ‘get stuck′ within IF. This tension seemed to have developed over time as IF evolved. A suggested solution to this from staff was to make participation as an IF member time limited, with the idea that this would provide greater focus on gaining independence and moving on from services (Table 4, Q16). However, this did not address the issue of IF members who benefited from engagement but needed some continuing support because of the effects of profound trauma.

### 3.6 Staff Survey Perspectives on Co‐Production

**Figure 4:**
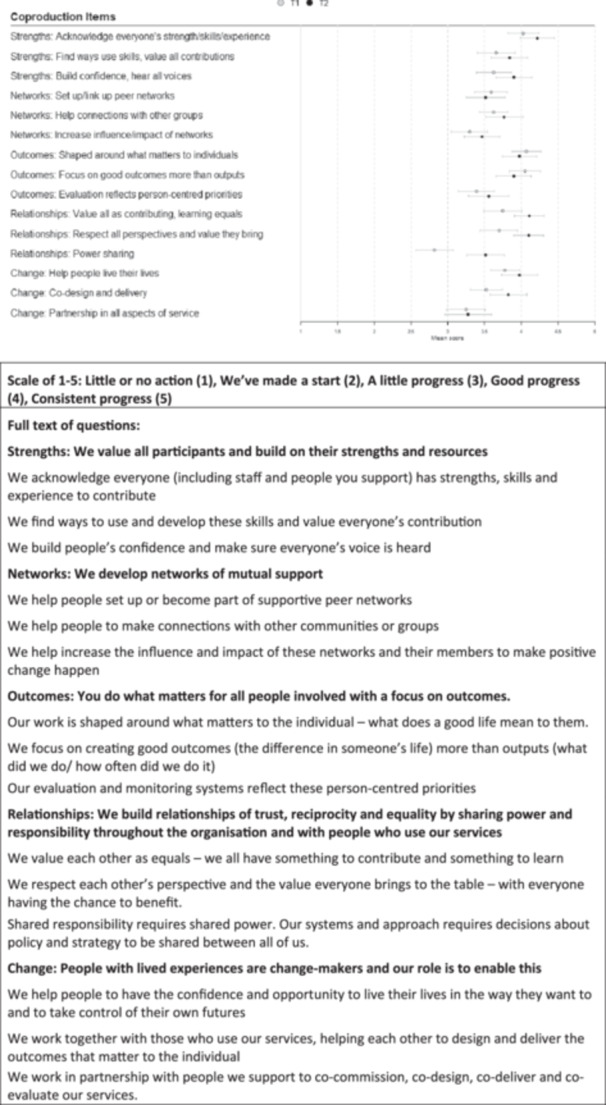
Co‐production audit questions (dots represent average score across all respondents, and bars are 95% confidence interval around the average).

To understand staff perspectives, a survey was distributed to staff within CF partner organisations, including voluntary and community sector organisations and adult social care services within the local authority. At time point 1 (T1) we received 85 responses, and a year later at time point 2 (T2) we received 62 responses (117 staff in total with 30 completing both surveys). Responses were collated for all organisations, as we received less than 10 responses from some organisations. The co‐production audit questionnaire [43] consisted of 15 action statements, as detailed in Figure 4. Client outcomes showed the strongest results:

- ‘Our work is shaped around what matters to the individual’ (average 4.1 at T1 and 4.0 at T2).

- ‘We focus on creating good outcomes—the difference in someone's life’ (average 4.1 at T1 and 3.9 at T2).
- Acknowledging everyone's strengths and skills (average 4.0 at T1 and 4.2 at T2).

The lowest average ratings were around shared responsibility and power at a policy level at T1; this improved by T2 but increases were not statistically significant:

- Decisions about policy and strategy are shared amongst all (average 2.8 at T1 and 3.5 at T2).

Staff free text comments illustrated a commitment to more co‐productive ways of working, with some areas of good practice, dependent on resources and capacity (Table 5, Q17–19).

**Table 5:** Broader staff perspectives from survey.

No.	Quote text	Trauma‐informed principles (*implementation domains where appropriate*)
Q17	‘*Our ability/commitment to co‐production very much depends on the budget, contract requirements and capacity within individual teams/services. It′s an aim/aspiration/goal for all services but there are significant variances between services/portfolio areas’.* (Staff survey 24T1)	Empowerment *(Financing, Monitoring and quality assurance, i.e., is co‐production included within contract requirements)*
Q18	‘*Resources still restrict this working better. Money, time, staffing, support networks always lacking’* (Staff 78T2)	Peer support, that is, support networks, collaboration *(Financing, workforce development)*
Q19	‘*(Name) is a top down organisation. Almost all change is top down and with very minimal and meaningless collaboration and consultation’.* (Staff survey 63T1)	(Lack of) collaboration *(Governance and leadership)*

Qualitative comments illustrated that collaborative managerial approaches alongside resources were enablers to co‐production. In contrast, hierarchical cultures with little sense of involvement were barriers (Table 5, Q18). The staff survey depicted a nuanced complexity where it was difficult to embed co‐production more broadly within all CF partners. There was a contrast of experiences, sometimes within the same organisation. Sufficient capacity, staff expertise in how to manage co‐production, appropriate support for people with lived experience and being able to engage with current service users were seen as important aspects to build on. Staff more often referred to barriers in relation to the implementation domains of TI practice, for example, financing, governance and leadership, and workforce development, with the associated lack of key TIPs around collaboration, peer support and empowerment. Whilst CFB had specific programme funding until March 2025 to enable IF to exist to support services and systems change, other services were much more stretched in their capacity and resource.

### 3.7 Future of IF

Towards the submission of this article, IF was changing to focus on sharing learning across a variety of organisations. There were plans to develop the range and diversity of co‐production initiatives across the city, building on learning from IF and the work of other existent lived experience groups within the area, enabling and supporting best practice.

## 4 Discussion

Co‐production needs to be tailored to meet the needs of people who experience MD [17, 18]. TIPs may support this, with some evidence of positive outcomes [7, 19]. This article provides evidence of how TIPs can be operationalised to establish safe relationships, sustained engagement over time, alongside training and development opportunities, to develop interpersonal and professional skills for effective co‐production. Table 6 illustrates how TIPs can be applied consistently within lived experience involvement and that lived experience involvement also needs to operate within the different implementation domains so that services can become more trauma‐informed. Table 6 illustrates how all TIPS are important, and it is how they interweave together that makes an environment more trauma‐informed.

**Table 6:** The embedding of TIPs and implementation domains to overcome barriers to co‐production within MD.

**Trauma‐informed principles**	**How does the enactment of TIPs and work within implementation domains address barriers to involvement for people with MD**
Safety	Safety was foundational to building trust and positive relationships with people with MD, enabled by other TIPs in practice (particularly transparency and trust, empowerment and choice, awareness of specific cultural, gender and historical issues, and peer support to connect with others). From the start IF members were encouraged to prioritise and take responsibility for their well‐being. Individual support, actions, interactions, meetings and plans were nuanced with flexibility. IF staff needed the ability to hold challenge and disagreement. This required humility on all sides and could be difficult to navigate. IF staff demonstrated ‘unconditional positive regard′ [47] irrespective of the members history, whilst maintaining boundaries by addressing any unacceptable behaviour in private. The purposeful development of safe relationships prioritised individual well‐being and encouraged thinking about and considering others in actions and relationships. The consistent demonstration of TIPs by staff over time served to increase the sense of safety, which enabled IF members to develop trust in the organisation.
Peer support	Enabled positive peer relationships and helped to support IF members with challenges. Supported people to be aware of the consequences of ‘over‐sharing′ and how this may cause distress to other members.
Trustworthiness and transparency	Clear boundaries were explained and enacted. Required standards were agreed with IF members, such as: punctuality; listening skills; considered responses to others; monitoring one's emotions; and a cancellation policy. Consistency and transparency in communication engendered trust. IF staff ensured conscious efforts were made to create and maintain trusting relationships with new members before any collaborative working (or work involvement) took place. Transparency in decision‐making was demonstrated wherever possible. Lived experience feedback was acknowledged and responded to where possible. Where this was not possible, this was explained. Staff did not hide behind their role, for example, staff member shared aspects of their own lived experience to highlight common humanity and demonstrate vulnerability whilst maintaining dignity and boundaries.
Collaboration	Collaboration was supported by IF staff, who offered choice, preparation sessions and debriefs as individually required.
Empowerment and choice	Flexibility in ways to engage with IF meetings helped to mitigate differing levels of wellness and recovery, enabling a sense of safety. Taking time away from IF when needed was encouraged, illustrating the need for fluidity and flexibility in involvement. Flexibility in ways to engage and being able to take ‘time out′ to prioritise self‐care was crucial to maintain engagement over time and cumulative progress. IF members were encouraged to work ‘slightly outside their comfort zone′, to improve resilience by adaptation, with the usual ‘no pressure′, ‘when you′re ready′ approach, maintaining choice as a TIP. When preparing to present ideas, or deliver training externally, there was always a backup plan to reduce pressure on members, who reported that they achieved a sense of satisfaction by collaborating and contributing to something that may benefit others. Members reported personal self‐development, such as feeling greater control of their personal narrative and direction in life, moving away from harmful people and environments, and growing in compassion for others.
Cultural, historical and gender issues	Options for appropriate safe spaces were ensured dependent upon specific needs of diverse populations. For example, when new IF members who had experienced domestic abuse joined, the option of women‐only spaces was explored with existing and new members. New members did not express a need for a separate women‐only space but appreciated the offer. This proposition remained available in case circumstances changed. Interviewees acknowledged that whilst this may have been necessary for women earlier in their recovery journey, they were sufficiently stable to work with men. The new members felt safe in the mixed‐sex group; a need for a women‐only space did not arise.
**TI implementation domains**	**What work is needed to address barriers to involvement for people with MD?**
Governance and leadership	IF had a collaborative and empowering management style. Management, practice and support within IF was explicitly, consistently driven and reflected on, using TIPs. Transparency and clarity in decision‐making (most of the time) Staff were responsive to feedback Horizontal power dynamics challenged hierarchical structures and were maintained by navigating relationships according to TIPs.
Policy	Having spaces to feed into policy, as highlighted in Figure 3, enabled IF to be able to influence at both local and national policy levels. The openness of governance and leadership to this potential was essential to be able to create these spaces.
Physical environment	IF members spent time creating their own safe spaces and rooms, for example, decorated with their own art. Discussions on how spaces needed to be designed to ensure safety were explicit, inclusive and acted upon.
Engagement and involvement	Supporting people with lived experience to carefully manage how they share their own experiences, helped to avoid flashbacks and the reactivation of trauma. A proactive and successful recruitment drive to broaden membership was undertaken with the involvement of current members.
Cross‐sector collaboration	When members felt ready, they were supported to engage and collaborate with partner organisations. This support enabled IF members to get involved with a large range of activities and developments (Figure 3), illustrating how traditional systems and structures could be challenged and changed.
Screening, assessment and treatment services	Appropriate support, using a flexible and personal ‘well‐being first′ approach for people with lived experience meant they could manage their own involvement, whilst also managing other challenges in life.
Training and workforce development (including here the recruitment, induction and training with people with lived experience)	Assurances of safety, trustworthiness and choice were essential at recruitment and induction of lived experience members. IF members were introduced to TIPs at induction training which included: avoiding oversharing, good listening skills, staying within your comfort zone, taking timeout, seeking help and safe behaviors for self and towards others. Practical skills training was also provided. IF meetings, training and well‐being sessions provided opportunities to practice effective communication and the creation of safe relationships, making collaboration possible and sustainable. Reflective practice was facilitated. Structured support helped to build personal and professional capacity with resilience. Staff support to enable and facilitate appropriate lived experience involvement.
Progress, monitoring and quality assurance	Immediate feedback to people with lived experience of how their involvement made a difference was appreciated. More formal and regular monitoring of lived experience involvement within services is required to support integration and avoid tokenism.
Financing	Funding stability to ensure long‐term lived experience involvement is key. However, this was difficult to enable in practice, in the long term. To make co‐production more sustainable, all service commissioning could include designated budgets for co‐production. This would help to support its embedding and meaningful long‐term initiatives, rather than relying on additional, small, time‐limited pots of money.
Evaluation	Growing the evidence base around best practice and impact in co‐production could support more funding.

### 4.1 TIPs as Personal Values and Organisational Norms

Organisations may ‘sign up’ to operating on the basis of TIPs, but without staff committing to these principles as personal values, it may appear tokenistic [18]. Staff within an organisation may or may not value TIPs as guides to behaviours, or they may value them but be compromised in their ability to always act accordingly and consistently because of stress, overwork, time pressures, their own trauma experiences, difficult relationships or feeling unsupported. Personal commitment to the values inherent in TIPs, supported by organisational norms and structures, that included staff and IF members alike, appears to have been the driving force behind IF success. Decisions and interactions, whether planned or ‘in the moment′, aligned with one or more TIPs. This commitment to TIPs avoided tokenism, and the mutual trust that developed was essential for co‐production to work.

Power dynamics (including organisational culture and leadership style) are particularly important when working alongside people with experience of MD and trauma [18]. Often, people will have first hand experience of powerlessness or misuse of power and are likely to be sensitive to power imbalances as an indicator of their vulnerability in an unsafe situation, based on past experiences [17, 18]. Several strategies within IF enabled a horizontal power dynamic, as highlighted within Table 6. However, given the aggregate amount of trauma, with people at different stages in recovery, and ongoing day‐to‐day challenges, it was inevitable that some tensions and struggles would emerge. An emphasis on safe relationships, good listening skills and accompanying respect for self and others supported resolution of disagreements. The TI approach, demonstrated by IF staff and members, is a microcosm of good practice at the grassroots level. However, the horizontal power dynamic and degree of safety was not always experienced when working with partner organisations, where commitment to co‐production and TIPs may not have been embedded, despite stated intentions. The staff survey illustrates a complex picture of the difficulties in extending co‐production and TIPs across multiple services. Hierarchical structures, cultures and resource pressures limited the extent to which co‐production was a reality for staff within CF partner organisations.

### 4.2 Implications for Theory and Practice

The national evaluation of the Changing Futures programme highlights that ‘there is still more work to be done to ensure that people with lived experience are involved consistently and meaningfully across sectors and services’ [48, p. 37]. Our findings are vital learning points for others to achieve this and align with previous work in creating systems change in MD [25]. IF members highlighted how successful involvement in co‐production may support ‘post‐traumatic growth’, where members could feel an increased sense of personal strength, a change in perspective, stronger connection with others and an identification of new opportunities [49]. However, the extent to which this then enabled progression beyond IF and further career development [2] is not clear from this time‐limited data.

Whilst staff were motivated to work more co‐productively in survey comments, barriers cited echo existing literature, such as difficulties of time, resources, leadership and organisational structures [17, 18]. For meaningful co‐production, all decision‐making levels need to be more participatory [18]. Personal and organisational commitment to the values inherent in TIPs, supported by cultural norms, is highlighted by this study. There is a need to work with people with lived experience to identify strategies and solutions to address trauma [26] and whilst there is increasing policy attention on this [1, 2], it is necessary to tackle these broader cultural, structural and resource barriers. There is a lack of infrastructure support for systems change within MD, with a need for more lived experience involvement [50]. The previous Fulfilling Lives MD programme highlighted that funding stability and relationship building were essential [51], as has been highlighted in other work [27]. Uncertain short‐term funding and the need to connect with other lived experience forums to bring wider perspectives has meant IF has been unable to remain in its current form. CFB's focus is now on embedding co‐production and collaborating between professionals and existing lived experience groups to facilitate co‐production initiatives. There are also plans to build on learning from IF to develop a lived experience alliance to link all services and organisations interested in co‐production.

### 4.3 Strengths and Limitations

Our study was strengthened by being collaborative from the outset with lived experience involvement in the funding bid, research design, analysis, reviewing literature on co‐production, and regular writing meetings. We achieved breadth and depth of data by interviewing 16 of 17 current members, and one interview with a previous member. Interviewees had been involved with IF from 3 months to 9 years, capturing historic and new perspectives. Staff most directly involved with IF also provided interviews. Recruitment and conduct of interviews also conformed to TIPs. However, this may have restricted the data collected in favour of respecting the privacy of the interviewee. IF members in the writing group agreed that the approach taken was appropriate. The study is limited to one Changing Futures area; however, findings align with and extend the national evaluation of the Fulfilling Lives programme [25], by illustrating how the embodiment of TIPs into practice across TI implementation domains can open up co‐production processes to people who have experienced MD.

## 5 Conclusion

IF has delivered a trauma‐informed model to enable personal and professional development for people who've experienced MD so that they can facilitate changes in policy and practice (Figure 3). IF has put TIPs into co‐production as a holistic approach. This enabled both IF members and staff to maintain personal and professional well‐being. Translating principles into practice can enable people with lived experience of MD to find and use their authentic voice for positive change, which can be life‐changing. This has been possible because TIPs are not a prescribed ‘tick‐list′ of requirements but rather an ethos that is authentically subscribed to by IF members and staff alike. TIPs are effective in enabling a positive and constructive working environment, if they apply to both staff and people with lived experience of MD. Any evidence of tokenism destroys trust and undermines the endeavour. Further work and longer‐term funding is needed to spread and embed these principles over the wider system for both staff and lived experience members alike. An authentic approach to co‐production and trauma‐informed practice needs to happen through all levels of the system for transformational change.

## Author Contributions

**Tracey Stone:** conceptualization, methodology, investigation, formal analysis of qualitative data, writing original draft, writing review and editing. **Emily Eyles:** conceptualization, methodology, investigation, formal analysis of the quantitative data, writing original draft, writing review and editing. **Thomas Traub:** conceptualization, methodology, formal analysis, writing original draft, writing review and editing. **Jason Burrowes:** conceptualization, methodology, formal analysis, writing review and editing. **Rebecca Halsley:** formal analysis, writing original draft, writing review and editing. **S.J.M.:** formal analysis, writing original draft, writing review and editing. **Joanna Gillam:** formal analysis, writing original draft, writing review and editing. **Maria Theresa Redaniel:** conceptualization, methodology, writing review and editing. **Sabi Redwood:** conceptualization, methodology, writing original draft, writing review and editing. **Corrado Totti:** conceptualization, methodology, writing review and editing. **Tania Smith:** investigation, formal analysis, writing review and editing. **Michelle Farr:** principal investigator, conceptualization, methodology, formal analysis of qualitative data, writing original draft, writing review and editing.

## Ethics Statement

The University of Bristol Faculty of Health Sciences Research Ethics Committee approved the research, reference 12277. The local authority involved also approved the study, BNSSG ICB reference: 2022‐087 and BCC reference: 2022‐020.

## Consent

All interview and survey participants completed informed consent procedures. All participants gave consent to participate and to publish anonymised quotes, in a written format for in‐person interviews and surveys and in a verbal recorded format for online/phone interviews before the interview/survey began.

## Conflicts of Interest

The author(s) declared the following potential conflicts of interest with respect to the research, authorship and/or publication of this article. Corrado Totti, Joanna Gillam and Tania Smith are staff of Changing Futures Bristol and Independent Futures. Thomas Traub, Jason Burrowes, Rebecca Halsley and S.J.M. are members of Independent Futures. Independent Futures received funding via the NIHR Three Research Schools Mental Health Practice Evaluation Scheme Grant Reference Number: MH021 for their time to get involved to co‐produce this study. All other authors report no potential conflicts of interest with respect to the research, authorship and/or publication of this article. University of Bristol authors collected data on IF member experiences to maintain independence and anonymity, and IF members were involved in anonymised analysis after an initial, independent analysis had been conducted by University of Bristol authors.

## Data Availability

Data associated with this manuscript is accessible only to the research team and is not publicly available due to concerns about confidentiality in a study based in an identifiable organisation, with a specific locality and small sample size.
